# Performances of full cross-validation partial least squares regression models developed using Raman spectral data for the prediction of bull beef sensory attributes

**DOI:** 10.1016/j.dib.2018.04.056

**Published:** 2018-04-23

**Authors:** Ming Zhao, Yingqun Nian, Paul Allen, Gerard Downey, Joseph P. Kerry, Colm P. O’Donnell

**Affiliations:** a School of Biosystems and Food Engineering, University College Dublin, Belfield, Dublin 4, Ireland; b Department of Food Quality and Sensory Science, Teagasc Food Research Centre, Ashtown, Dublin 15, Ireland; c School of Food and Nutritional Sciences, University College Cork, Cork, Ireland

**Keywords:** Selected Raman shift ranges, Sensory attributes, Bull beef, Partial least squares regression models

## Abstract

The data presented in this article are related to the research article entitled “Application of Raman spectroscopy and chemometric techniques to assess sensory characteristics of young dairy bull beef” [1]. Partial least squares regression (PLSR) models were developed on Raman spectral data pre-treated using Savitzky Golay (S.G.) derivation (with 2nd or 5th order polynomial baseline correction) and results of sensory analysis on bull beef samples (*n* = 72). Models developed using selected Raman shift ranges (i.e. 250–3380 cm^−1^, 900–1800 cm^−1^ and 1300–2800 cm^−1^) were explored. The best model performance for each sensory attributes prediction was obtained using models developed on Raman spectral data of 1300–2800 cm^−1^.

## Specifications Table

**Table:** 

Subject area	*Spectroscopy, Chemometrics*
More specific subject area	*Performance of PLSR models developed using selected Raman shift ranges (i.e. 250–3380 cm^−1^, 900–1800 cm^−1^ and 1300–2800 cm^−1^)*
Type of data	*Table*
How data was acquired	*Raman spectroscopy, Results of sensory analysis, Chemometrics*
Data format	*.doc*
Experimental factors	*Raman spectral data were pre-treated using Savitzky Golay (S.G.) derivation with 2nd or 5th order polynomial baseline correction.*
Experimental features	*–*
Data source location	*School of Biosystems and Food Engineering, University College Dublin, Belfield, Dublin 4, Ireland*
Data accessibility	*Data is with this article*

## Value of the data

• To demonstrate PLSR models developed using Raman spectra in the 1300–2800 cm^−1^ range can give best prediction performance on sensory attributes of bull beef.
• Results of this work are in agreement with a previous study by Nian et al. [2] that the Raman frequency range of 1300–2800 cm^−1^ is the most suitable range for prediction of bull beef eating quality parameters.
• This data suggested other researchers to select an optimal Raman shift range for further meat science studies.

## 1 Data

PLSR models were developed on Raman data pre-treated using Savitzky Golay (S.G.) derivation with 2nd and 5th order polynomial baseline correction. Prediction performance of models developed using selected Raman shift ranges (i.e. 250–3380 cm^−1^, 900–1800 cm^−1^ and 1300–2800 cm^−1^) were summarized in Table 1. PLS models developed using S.G. derivation pre-treated Raman spectra in the 1300–2800 cm^−1^ range performed best (R^2^CV values of 0.36–0.84) while spectra in the range 900–1800 cm^−1^ performed worst (R^2^CV values of 0.03–0.66). Results shown in this work are the supplementary materials of the research article ׳Application of Raman spectroscopy and chemometric techniques to assess sensory characteristics of young dairy bull beef׳ [1].

**Table 1:** Summary of full cross-validation PLSR model performances for sensory attributes prediction using all beef samples (*n* = 72).








PLSR, partial least squares regression models; S.G., Savitzky Golay; der., derivatives; nor.u.v., normalisation on unit vectors; # PLS loadings, number of PLS loadings; R^2^C, coefficient determination of calibration; RMSEC, root mean square error of calibration; R^2^CV, correlation coefficient of determination in cross-validation; RMSECV, root mean square error of cross-validation; IT, Initial Tenderness; ED, Ease of Disintegration; Res-, Residual (after effects); *n*, numbers of samples. (Note: The best performed PLS models developed in the Raman shifts of 1300–2800 cm^−1^ were highlighted in yellow).

## 2 Experimental design, materials and methods

For the prediction of beef sensory attributes, partial least squares regression (PLSR) models were developed using pre-processed Raman spectroscopic data (X data) collected on the 21st day post-mortem using pre-selected frequency ranges (i.e. 250–3380 cm^−1^, 900–1800 cm^−1^, 1300–2800 cm^−1^); these were selected on the basis of spectral signal intensities. Measured values of sixteen sensory attributes were used as individual Y variable for PLS regression. Leave-one-out cross-validation was performed to evaluate the performance of PLSR models using parameters such as root mean square error of calibration (RMSEC) and cross-validation (RMSECV), the coefficient of determination on calibration (R^2^C) and cross-validation (R^2^CV) and the bias which is calculated as the difference between the average of actual and predicted values for each data set [3]. For a satisfactory prediction performance, the value of *R*^2^ is expected to be close to 1 while values of RMSECV and bias are expected to be close to 0.
