# Supplementary material for: Performances of full cross-validation partial least squares regression models developed using Raman spectral data for the prediction of bull beef sensory attributes
Source: Data Brief. 2018 Apr 23;19:1355–60. doi: 10.1016/j.dib.2018.04.056 (PMC6141602; doi:10.1016/j.dib.2018.04.056)
Supplement: Supplementary file 1 — Supplementary material [file mmc1.docx]

Conflict of Interest Form

This project was submitted to the Teagasc Animal Ethics Committee which advised that since best husbandry practice was followed no ethical issues arose.

No conflict.
